# Assessment of Place of Delivery and Associated Factors among Pastoralists in Ethiopia: A Systematic Review and Meta-Analysis Evaluation

**DOI:** 10.1155/2023/2634610

**Published:** 2023-11-09

**Authors:** Lebeza Alemu Tenaw, Henok Kumsa, Mulugeta Wodaje Arage, Atitegeb Abera, Tilahun Hailu, Esuyawkal Mislu

**Affiliations:** ^1^School of Public Health, College of Health Science, Woldia University, Ethiopia; ^2^School of Midwifery, College of Health Science, Woldia University, Ethiopia

## Abstract

**Background:**

Pastoralist communities rely on their livestock for at least 50% of their food supply and source of income. Home births raise the risk of maternal morbidity and death, whereas institutional births lessen the likelihood of difficulties during labor. Around 70% of labors in pastoralist regions of Ethiopia were assisted by traditional birth attendants.

**Methods:**

Studies done from January 2004 to January 2023, accessed in PubMed, EMBASE, Medline, and other search engines, were included. PRISMA guidelines and JBI critical appraisal checklist were used to assure the quality of the review. Ten articles were included in this review. Data were extracted with Excel and exported to STATA 16 for analysis. Heterogeneity of literatures was evaluated using *I*^2^ statistics and publication bias using the Egger regression asymmetry test and the Duval and Tweedie trim-fill analysis. Statistical significance was declared at *p* value less than 0.05.

**Result:**

The pooled estimate of institutional delivery among the pastoralist community in Ethiopia is 21.2% (95% CI: 16.2-26.1). Husbands who were involved to decide place of delivery (OR = 3.47; 95% CI: 1.61, 7.50), women with good knowledge of MCH services (OR = 2.283; 95% CI: 1.51, 3.44), women who had a positive attitude towards MCH services (OR = 1.69; 95% CI: 0.79, 3.6), availability of health institutions (OR = 2.6; 95% CI: 0.95, 7.20), and women who had an ANC follow-up (OR = 2.78; 95% CI: 2.07, 3.73) were higher institutional delivery prevalence among pastoralist women. Moreover, institutional delivery among women who were educated above the college level was more than two times (OR = 2.56; 95% CI: 1.985, 3.304) higher than among women who were not educated.

**Conclusion:**

Pastoralist women in Ethiopia were found to be a disadvantaged group for institutional delivery at national level. Husband involvement, educational level, ANC visit, knowledge and attitude for MCH service, and health facility distance were identified to have significant association with institutional delivery.

## 1. Introduction

Pregnancy is a physiological state that most women aspire to have at some point in their lives [1]. Women can give birth in either a health facility or at home, which are mutually exclusive options. Home births raise the risk of maternal morbidity and death, while institutional births ensure a safe delivery, lessen the likelihood of difficulties during labor and shortly after delivery, and increase the survival of mothers [2–7].

Reducing maternal mortality remains a priority agenda under goal three in the UN Sustainable Development Goals (SDGs) through 2030 [8]. Globally, it continues to be a significant public health issues, particularly in sub-Saharan Africa. The maternal mortality ratio (MMR) has decreased by nearly 44% over the past 25 years, with an estimated 216 maternal deaths per 100,000 live births in 2015, compared to a MMR of 385 in 1990. However, the incidence of maternal mortality remains unacceptably high in developing countries, which account for 99% of global maternal deaths [9–13]. The approximate global lifetime risk of a maternal death has significantly decreased from 1 in 73 to 1 in 180 [14].

The presence of qualified health care providers during delivery is the most effective strategy for reducing maternal mortality worldwide and serves as an indicator to monitor national efforts towards safe motherhood [15]. However, the proportion of skilled delivery is low in southern Asia and sub-Saharan Africa (SSA), with rates of 40% and 47%, respectively [16]. Ethiopia, one of the sub-Saharan African countries, has a high maternal mortality ratio [17–19], in which only 15% of births are delivered at a health facility [20, 21]. As of 2016, the estimated maternal mortality ratio in Ethiopia was 412 per 100,000 live births [21].

There are numerous kinds of pastoralism all around the world. Communities considered to be pastoralists rely on their livestock for at least 50% of their food supply and source of income, which is considered to be the norm. In sub-Saharan Africa, the size of the pastoralist population is estimated to be around 50 million, out of which 20 million are found in Ethiopia, Eritrea, Djibouti, Somalia, and Uganda. About 12% (12–15%) of Ethiopia's population, covering 61-63% of the country's land mass, are pastoralists. The pastoral areas are estimated to comprise 42% of the national total livestock population, which contributes a lot to the national economy [22, 23].

Different studies revealed that nomadic pastoralist populations have difficulty to access reproductive and maternal health care services despite their preference to use it [24]. They are among the most underserved and hard-to-reach populations [25–27]. The main reasons were physical locations (distant health facilities) and political, economic, and sociocultural contexts [25, 26]. In nomadic pastoralist population, maternal death is more than two times higher among women who delivered at home [26].

The overall rate of institutional delivery practices among pastoralists in Ethiopia is significantly lower compared to the national level [28]. Reports from the Ethiopia Demographic and Health Survey (EDHS) 2016, as well as other studies conducted in Ethiopia, have shown that the utilization of skilled delivery services provided by skilled birth attendants (SBAs) is extremely low in pastoral areas. Additionally, more than 70% of women in predominantly pastoralist regions of Ethiopia receive assistance from traditional birth attendants (TBAs) at home [29–31]. This results in a higher rate of maternal death among pastoralist populations [28–31].

The low rate of institutional delivery among pastoralists is primarily influenced by factors such as distance to health facilities, low coverage of antenatal care, and limited awareness about ANC follow-up and institutional delivery services [5, 21, 28, 32–41]. Additionally, factors such as previous history of stillbirth, adequate knowledge about pregnancy and childbirth, involvement of the husband in decision-making, socioeconomic status (measured by wealth index), educational attainment of mothers, type of health care facility, perceived quality of service, health insurance coverage, information about birth preparedness plans, respect for women's modesty, delivering babies while naked, and separation from family during delivery were found to be associated with delivery service utilization [5, 6, 21, 28, 32–45].

However, at the national level, Ethiopia lacks comprehensive data on the choice of delivery site and factors that influence place of delivery. To address this gap and determine the prevalence of institutional delivery and the factors influencing it in Ethiopia, a systematic review and meta-analysis were conducted. The findings of this study provide valuable support for efforts to reduce the burden of home delivery, including its complications and impact on the country. Additionally, the results of this study will contribute to the improvement of institutional delivery strategies and the monitoring of progress towards achieving the targets set by the Sustainable Development Goals (SDGs) for reducing maternal mortality.

## 2. Methods

**Figure 1:**
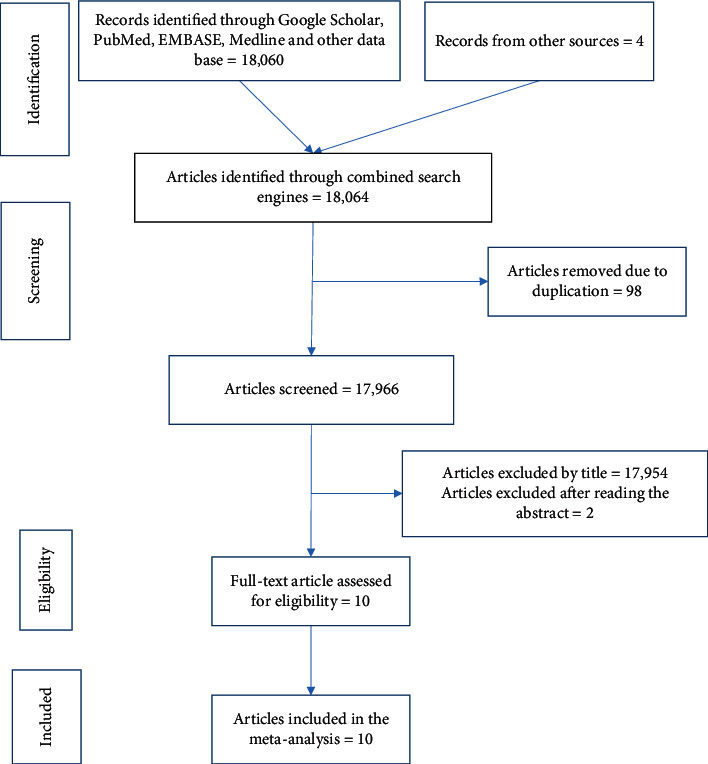
Flow chart of study selection for systematic review and meta-analysis of institutional delivery and associated factors among pastoralist communities in Ethiopia.

This systematic review and meta-analysis were conducted to identify the pooled prevalence of institutional delivery (outcome variable) and associated factors (exposure variables) among pastoralist community in Ethiopia. The flow chart for article selection is shown in Figure 1.

### 2.1. Searching Strategy

International databases (Medline/PubMed, Google Scholar, Cochrane Library, and Google Scholar), different gray pieces of literature, and articles published or unpublished in the university's online repository were included in this review. The search terms were as follows: (((((((((((Prevalence) OR (magnitude)) OR (level)) AND (associated factors)) OR (predictors)) OR (determinant factors)) AND (institutional delivery)) OR (home delivery)) OR (health facility delivery)) AND (pastoralist)) OR (pastoral community)) AND (Ethiopia).

### 2.2. Inclusion and Exclusion Criteria

The inclusion criteria were observational studies (cross-sectional and case-control) that revealed the prevalence of institutional delivery and/or had at least one factor associated with the outcome variable. Only English-language literature and research articles were considered. Articles that lacked sufficient information to calculate accurate risk estimates and the corresponding 95% confidence interval, as well as those with no full abstracts or full-text versions, were excluded.

### 2.3. Quality Assessment

Literature and articles were screened using their titles, abstracts, analysis and ways of reporting results, and full papers before they were included in the meta-analysis. The quality of the included studies was evaluated using the Joanna Briggs Institute (JBI) critical appraisal checklist [46]. The quality scores of the included studies were assessed and presented using the mean scores to designate them as high or low quality. Of all 8 questions in the JBI criteria, studies that had a score of 5 or more were considered to have good quality and included in the review. Any discrepancies in critical appraisal among reviewers were resolved through discussion with the third person reviewer. Additionally, Preferred Reporting Items for Systematic Reviews and Meta-Analysis (PRISMA) checklist is also filled to show content-related quality of the report (Supplementary Table 1).

### 2.4. Data Extraction and Management

After the data had been screened by title and abstract and reviewing the articles, three authors (*EM*, *TH*, and *LAT*) independently reviewed the titles and abstracts of studies to be included and agreed on the articles included in this systematic review and meta-analysis. The data were prepared/extracted in Microsoft Excel form and checked and evaluated by all authors. The JBI data extraction tool contains information on the author and year of the study, the title, the year the study was conducted and the year of publication, the study area and region, the study design and type, the study population, the sample size, the outcome measured, and the prevalence and predictors of institutional delivery [47].

### 2.5. Registration and Protocol

This review had no a registered protocol. As a result, no adjustment has been made.

### 2.6. Data Analysis

The data were analyzed using STATA version 16.0. The characteristics of the original studies were described using tables and a forest plot. The heterogeneity between the primary studies was checked using *x*^2^ test and *I*^2^ test. Publication bias was checked using the funnel plot and Egger's regression test. Subgroup analysis was conducted using the study region and year of publication. The forest plot format was used to present the pooled point prevalence with 95% confidence interval (CI).

## 3. Results

### 3.1. Characteristics of the Articles Included in This Review

This review included the studies conducted from 2012 to 2023. The majority of the pastoralist community was found in Afar region (seven articles included in this review) [5, 28, 33, 34, 37, 45, 48], Somali region (one study included) [49], Oromia region (one study included) [43], and South Ethiopia (one article included) [44]. All studies included in this review were observational [5, 28, 33, 34, 37, 43–45, 48, 49] (Table 1).

### 3.2. Prevalence of Institutional Delivery among Pastoralist Community

**Figure 2:**
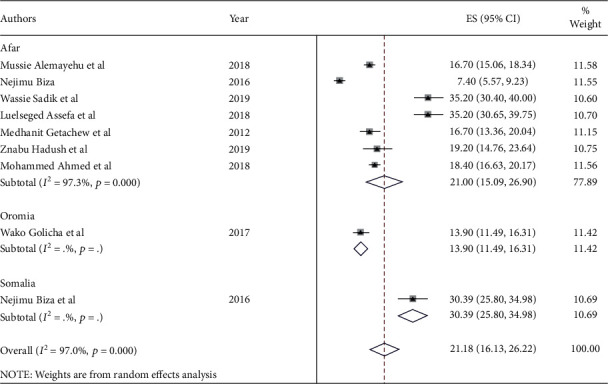
Overall prevalence of institutional delivery based on regional distribution among pastoralist community in Ethiopia, 2023.

**Figure 3:**
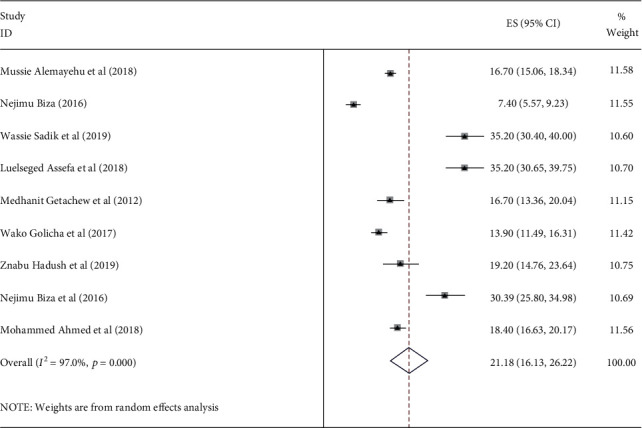
Forest plot of the pooled prevalence of institutional delivery among pastoralist community in Ethiopia, 2023.

**Figure 4:**
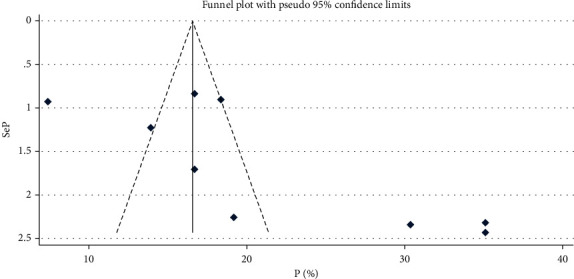
Funnel plot of publication bias among the studies done on prevalence of institutional delivery in Ethiopia, 2023.

**Figure 5:**
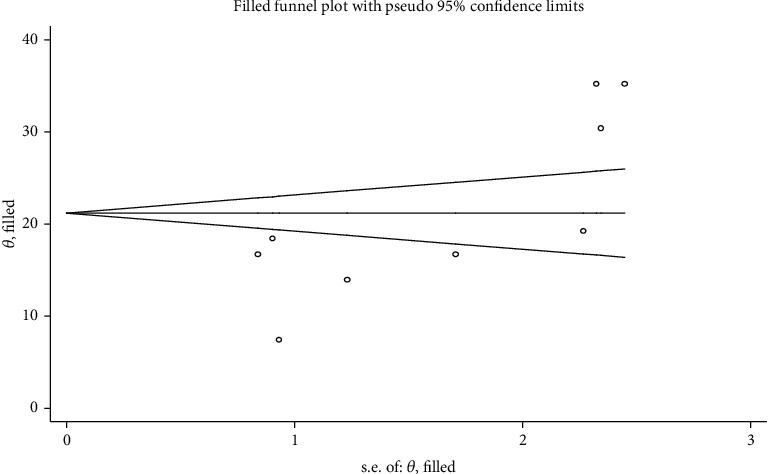
Trim and fill analysis of studies done on prevalence of institutional delivery in Ethiopia, 2023.

The prevalence of institutional delivery among pastoralist communities ranges from 13% in Oromia [43] to 21% in Afar [5, 28, 33, 34, 37, 45, 48] and 30.4% in Somalia [49] (Figure 2). The pooled estimate of institutional delivery among the pastoralist community in Ethiopia is 21.2% (95% CI: 16.2-26.1) [5, 28, 33, 34, 37, 43–45, 48, 49] (Figure 3). There is a significant level of heterogeneity (*I*^2^ = 98.0%; *p* value = 00) between the included studies, and based on Egger's regression asymmetry test, the study showed a significant level of publication bias (Figure 4). After adjustment, the pooled prevalence of institutional delivery among the pastoralist community after the trim and fill analysis was 21.2% (95% CI: 16.13–26.22) [5, 28, 33, 34, 37, 43–45, 48, 49] (Figure 5).

**Figure 6:**
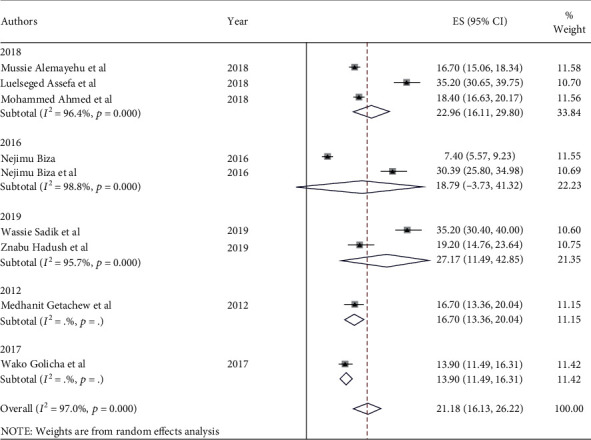
Overall prevalence of institutional delivery based on publication year distribution among pastoralist community in Ethiopia, 2023.

The subgroup analysis revealed that there is gradual improvement of utilizing health institutions for maternal health services in the pastoralist community. The overall estimate of institutional delivery during the year 2012 is 16.7% [33], which increases to 18.8% in 2016 [28, 49] and 27.2% in 2019 [5, 37] (Figure 6).

### 3.3. Factors Associated with Institutional Delivery among Pastoralist Community

Three articles were reviewed to determine the relationship between husbands' involvement in deciding where women should give birth [5, 44, 45]. The meta-analysis found that husbands who were involved in place of delivery preference were more than three times (OR = 3.47; 95% CI: 1.61, 7.50) more likely to deliver in a health institution than their counterparts. There was no significant heterogeneity between the studies (*I*^2^ = 59.6%, *p* value = 0.084). The pooled estimate of three articles revealed that women with good knowledge of MCH services were twice as likely (OR = 2.283; 95% CI: 1.51, 3.44) to deliver in a health institution than women with poor knowledge [5, 44, 45].

**Figure 7:**
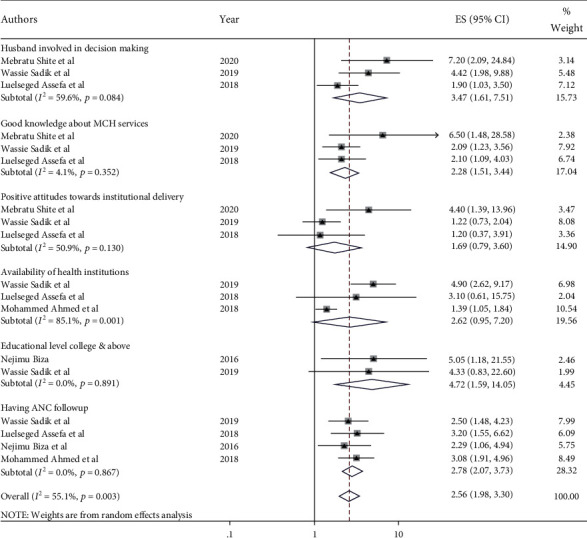
Factors associated with institutional delivery among pastoralist community in Ethiopia, 2023.

Based on the pooled estimate of three research articles [5, 44, 45], women who had a positive attitude towards MCH services had higher odds of institutional delivery prevalence (OR = 1.69; 95% CI: 0.79, 3.6) as compared to their counterparts. The availability of health institutions increases the odds of institutional delivery for the pastoralist community (OR = 2.6; 95% CI: 0.95, 7.20) [5, 45, 48]. The other four studies revealed that women who had an ANC follow-up had higher odds of institutional delivery prevalence (OR = 2.78; 95% CI: 2.07, 3.73) than those who had no ANC visit [5, 45, 48, 49]. Moreover, the meta-analysis of two articles showed that the prevalence of institutional delivery among women who were educated above the college level was more than two times (OR = 2.56; 95% CI: 1.985, 3.304) higher than among women who were not educated [5, 28] (Figure 7).

## 4. Discussion

Institutional delivery with skilled care provider is critical to decrease maternal and neonatal mortality and morbidity [14]. Due to this reason, worldwide, there is an increment of institutional delivery from 64% in 2001 to 84% in 2021 globally and from 40% in 2001 to 69% in 2021 among sub-Saharan countries [50]. Despite its benefit and improvement, different barriers hinder pastoralist women from accessing health care during labor and delivery. These barriers include geographical location, sociocultural dynamics, availability of logistics, political factors, norm and traditions, and individual perception. This made nomad pastoralist women experience completely different from the rest of the world [51].

This systematic review and meta-analysis identified that the pooled prevalence of institutional delivery in pastoralist community was 21.2% (95% CI: 16.2-26.1). This result is lower than the national pooled prevalence of institutional delivery which was 31% [52], and the 2019 national mini EDHS report 48% [53]. Even it is lower than the regional data which was 28.3% Afar, 23.3% Somalia, 41.0% Oromia, and 47.5% SNNP based on mini EDHS [53]. However, it is comparably similar with Somalia region 23.3% [53]. This finding showed that there is a great discrepancy to have institutional delivery between pastoralist communities and the rest of the population in Ethiopia [52, 53].

It is also found that this result is lower than studies done in rural nomads in rural western China 47.4% [54] and study conducted in nomad tribal population of Rajasthan, India 30% [55]. In the reverse, the pooled prevalence of institutional delivery in this meta-analysis was higher than a systematic review done among tribes of India which showed 17.7% [56].

Based on subgroup analysis, the overall estimate of institutional delivery increased from 16.7% by the year 2012 to 18.8% in 2016 and 27.2% in 2019. This increment is also found in the national meta-analysis which showed that the institutional delivery prevalence increased from 24% in 2011–2014 to 37% in 2017–2018 [52].

This meta-analysis identified that husband involvement on delivery place preference was significantly associated with an increase institutional delivery. This finding is supported with study done among mainly nomadic pastoralist population in Uganda [57] and Mali [58]. The other variable that was found to have significant association with increased prevalence of institutional delivery in this meta-analysis was having ANC visit. This result is in line with the finding of a literature review done in Ghana [59] and Ethiopia [52].

Knowledge on services given at MCH unit was found to have significant association with an increase in institutional delivery in this meta-analysis. This result is in line with the finding of a study conducted among pastoralists in Kenya [60], a review in Ghana [59], and studies done on sexual and reproductive health service utilization among nomads worldwide [51].

In this meta-analysis, knowledge on pregnancy danger signs and complications was identified to have positive association with institutional delivery. This result is supported by a finding of a literature review among pastoralists in Kenya [60], a systematic review of African pastoralists [27], and a study done among nomad in Sudan [61], Ghana [59], and Ethiopia [52]. Additionally, college and above educational level was identified to have significant association with institutional delivery among pastoralists in Ethiopia which is supported by reviews of studies done in Ghana which shows a high (90.9%) acceptance of institutional delivery among this population group [59]. It is also supported by a study done among nomad in Sudan [61], African pastoralists [27], and the general population of Ethiopia [52].

The other variable found to have significant association with institutional delivery in this meta-analysis was availability of health facility within a shorter distance. This is supported by a study done among pastoralists in Kenya [60], a literature review in Ghana [59], and a review of studies among nomads [51], African pastoralists [27], and tribes of India [56].

On interpreting this finding, it is important to consider its strength and limitations. As a limitation, in this study, variables like gestational age at initiation of ANC care, women preference for traditional birth attendants, involvement of woman on development army, presence of male health professionals in labor ward, history of still birth, availability of cash on hand at the time of labor initiation, delivery place for the most recent birth, and history of complication in the most recent birth were not entered for meta-analysis. This was because of heterogeneity of studies done to explain institutional delivery. As strength, this review tries to include all available studies on pastoralist population in Ethiopia.

## 5. Conclusion

This meta-analysis and systemic review identified that despite significant heterogeneity between studies, the prevalence of institutional delivery was too low among pastoralist women in Ethiopia as compared to the World Health Organization recommendation for universal coverage of institutional delivery practice, and pastoralist women in Ethiopia were found to be a disadvantaged group for institutional delivery at national level.

This review also identified that husband involvement on decision for delivery place choice, educational level, ANC visit, knowledge on danger sign and complications of labor and delivery, knowledge on services given at MCH unit, and health facility distance were identified to have significant association with institutional delivery. Therefore, it is better to consider the identified factors to improve institutional delivery in the pastoralist communities. Furthermore, large-scale interventional studies are needed to identify the detailed factors associated with institutional delivery and its impact on fetomaternal outcome.

## Figures and Tables

**Table 1:** Characteristics of studies included for systematic review and meta-analysis on institutional delivery among pastoralists in Ethiopia.

Authors	Year	Region	Study design	Study setting	Sample size	Prevalence in %
Mussie Alemayehu et al.	2018	Afar	Cross-sectional	Community level	1978	16.7
Nejimu Biza	2016	Afar	Cross-sectional	Community level	788	7.4
Wassie Sadik et al.	2019	Afar	Cross-sectional	Community level	381	35.2
Luelseged Assefa et al.	2018	Afar	Cross-sectional	Community level	423	35.2
Medhanit Getachew et al.	2012	Afar	Cross-sectional	Community level	478	16.7
Wako Golicha et al.	2017	Oromia	Cross-sectional	Community level	791	13.9
Znabu Hadush et al.	2019	Afar	Cross-sectional	Community level	302	19.2
Nejimu Biza et al.	2016	Somalia	Cross-sectional	Community level	385	30.39
Mohammed Ahmed et al.	2018	Afar	Cross-sectional	Community level	1842	18.4
Mebratu Shite et al.	2020	South Ethiopia	Case-control	Community level	292	—

## Data Availability

The datasets used and analyzed during the current study are available from the corresponding author upon reasonable request (esuyawkalmislu@gmail.com).

## Abbreviations

ANC: Antenatal care
CI: Confidence interval
EDHS: Ethiopia Demographic Health Survey
JBI: Joanna Briggs Institute
MCH: Maternal and child health
OR: Odds ratios
PRISMA: Preferred Reporting Items for Systematic Reviews and Meta-Analysis.

## Supplementary Materials

Supplementary Materials PRISMA 2020 checklist.
